# Determination of Ultrasound Reference Values for Diagnosing Low Muscle Mass in Older Chinese Adults

**DOI:** 10.1002/jcsm.70155

**Published:** 2025-12-08

**Authors:** Shumin Li, Keying Xu, Ange Wang, Chengfan Qin, Nan Hua, Xinrui Ling, Liqian Xu, Caihong He, Shixian Zhou, Jing Chen, Qin Zhang, Yunmei Yang

**Affiliations:** ^1^ Department of Geriatrics the First Affiliated Hospital, Zhejiang University School of Medicine Hangzhou China; ^2^ Zhejiang Key Laboratory for Diagnosis and Treatment of Physic‐Chemical and Aging‐related Injuries Hangzhou China

**Keywords:** biceps brachii, muscle mass, sarcopenia, tibialis anterior, ultrasound

## Abstract

**Background:**

Ultrasound is a promising tool for diagnosing sarcopenia, yet standardized cut‐off points and criteria are currently lacking. This study aimed to identify the optimal muscle sites and reference values for diagnosing low muscle mass in older Chinese adults using ultrasound, with dual‐energy X‐ray absorptiometry (DXA) as the reference standard, and to compare its diagnostic performance with bioelectrical impedance analysis (BIA).

**Methods:**

We included 1011 participants aged over 60 years. Fat thickness (FT), muscle thickness (MT) and muscle cross‐section area (CSA) at various sites, including biceps brachii, rectus abdominis, rectus femoris, vastus intermedius, vastus lateralis, vastus medialis and tibialis anterior, were assessed for participants. DXA measurements were used as the standard for defining low muscle mass. Receiver operating characteristic (ROC) curve analysis and 10‐fold cross‐validation were conducted to evaluate the prediction performance of ultrasound parameters for low muscle mass. The Youden index was employed to determine optimal cut‐offs, while sensitivity, specificity and accuracy were calculated to assess diagnostic performance. Intra‐class correlation coefficients (ICC) were used to evaluate inter‐rater reliability between two examiners.

**Results:**

In males, the biceps brachii CSA (AUC = 0.832, 95% CI: 0.793–0.870) and in females, the tibialis anterior MT (0.833, 95% CI: 0.789–0.877) demonstrated superior predictive power for low muscle mass compared with other ultrasound parameters. A biceps brachii CSA < 7.1 cm^2^ in males and tibialis anterior MT < 2.3 cm in females were identified as effective indicators for diagnosing low muscle mass, with good sensitivities (73.9% in males, 73.6% in females), specificities (78.6% in males, 76.8% in females) and accuracies (75.7% in males, 75.8% in females). These values were comparable with those obtained using BIA (sensitivity: 62.6%, specificity: 80.2% and accuracy: 71.5%). Low muscle mass as defined by ultrasound was significantly associated with poor performance on the Short Physical Performance Battery, Activities of Daily Living and frailty indices, with effect sizes higher than DXA or BIA defined low muscle mass. The inter‐rater reliability was excellent for biceps brachii CSA (ICC = 0.869, 95% CI: 0.650–0.937) and good for tibialis anterior MT (ICC = 0.730, 95% CI: 0.622–0.810).

**Conclusions:**

Muscle ultrasound demonstrated excellent inter‐rater reliability and stronger associations with adverse health outcomes compared with DXA and BIA, making ultrasound a preferable tool for assessing low muscle mass. This study provides valuable reference values for ultrasound‐based diagnosis of sarcopenia in older Chinese adults.

## 1 Introduction

Sarcopenia, an age‐related condition marked by a decline in muscle strength and function, significantly increases the risk of adverse health outcomes, including falls, disability and mortality [1]. The prevalence of sarcopenia among older adults worldwide has been estimated to range from 10% to 27%, with higher rates observed in hospital settings compared with community‐dwelling populations [2]. With the growing global older population, the number of individuals affected by sarcopenia is expected to rise. Early detection and diagnosis are crucial for effective prevention and treatment, improving outcomes and maintaining quality of life in older adults.

Assessment of muscle mass is necessary for the diagnosis of sarcopenia [3]. Computed tomography (CT) and magnetic resonance imaging (MRI) offer relatively precise measurements of both muscle quantity and quality. Nevertheless, their clinical application for sarcopenia assessment is limited because of CT's radiation exposure and the high cost of MRI [4]. Dual energy X‐ray absorptiometry (DXA) and bioelectrical impedance analysis (BIA) are widely recommended to evaluate muscle mass [3, 5]. Nonetheless, DXA is non‐portable and involves radiation exposure, while BIA measurements vary between different brands [5]. Moreover, DXA primarily quantifies fat‐free lean mass, which includes muscle but also other non‐fat tissues. While BIA estimates muscle mass based on the distribution of body water, under the assumption that muscle tissue has a distinct hydration status compared with other compartments. Both DXA and BIA methods provide indirect assessments of muscle mass rather than direct measurements [5].

Ultrasound is a portable, safe, cost‐effective imaging approach that can assess both muscle quantity and quality [6, 7, 8], making it a promising tool for sarcopenia diagnosis. More importantly, ultrasound could be easily accessible in primary care settings and could be used even in the intensive care unit for critically ill patients who are bedridden [9]. Studies have shown the potential validity of ultrasound in determining muscle mass and predicting clinical outcomes and functional performance [7, 10]. Muscle thickness (MT) and muscle cross‐sectional area (CSA) were the two most commonly used ultrasound parameters for assessing muscle mass [11, 12]. Previous ultrasound‐based assessments of sarcopenia have primarily targeted the quadriceps femoris muscles, including rectus femoris, vastus intermedius, vastus lateralis and vastus medialis, with attention also given to the biceps brachii, rectus abdominis and tibialis anterior [12, 13]. These muscles serve as representative sites across limb (both upper and lower extremities) and trunk regions and have been validated as reliable indicators for evaluating sarcopenia [12, 13]. However, studies generally using small sample sizes utilized different ultrasound parameters and muscle sites [11]. Thus, the optimal sites for diagnosing sarcopenia using ultrasound remain unclear. In addition, there are population‐specific ethnic variations for muscle mass. Currently, there is no consensus for diagnosing low muscle mass in older Chinese adults. Therefore, the determination of the optimal muscle site and the cut‐off points is important for promoting the application of ultrasound in diagnosing sarcopenia.

The current study aimed (1) to determine the optimal skeletal muscle sites and ultrasound reference value for diagnosing low muscle mass in older Chinese adults using DXA‐defined low muscle mass as standard, by measuring the MT and CSA of multi‐site muscle including biceps brachii, rectus abdominis, quadriceps femoris, rectus femoris, vastus intermedius, vastus lateralis, vastus medialis and tibialis anterior; (2) to compare the predictive and diagnostic performance of ultrasound‐defined low muscle mass against BIA‐defined low muscle mass; (3) to assess the association between low muscle mass defined by ultrasound parameters and health outcomes (SPPB, ADL and frailty) as compared with DXA and BIA‐defined low muscle mass; (4) to assess the inter‐rater reliability of ultrasound parameters for diagnosing low muscle mass.

## 2 Methods

### 2.1 Study Design and Participants

The cross‐sectional diagnostic study was conducted between October 2022 and September 2024. Participants were recruited from a teaching hospital and several community health service centres in Hangzhou, China. Recruitment strategies included the use of poster advertisements and health education sessions. The inclusion criteria were participants aged 60 years and above. Individuals with the following history were excluded: peripheral oedema, limb fracture or limb surgery within the past 6 months, diseases affecting the musculoskeletal system, acute critical illnesses, stroke resulting in limb hemiplegia, or diagnoses of Parkinson's disease or dementia. Additionally, those who were unable to comply with the testing procedures were also excluded. Each participant was informed of the study process and signed the consent before the implementation. The study was approved by the Clinical Research Ethics Committee of the first affiliated hospital of Zhejiang University School of Medicine (approval number: 2022‐966).

A total of 1041 participants were recruited for the study. Of these, 871 participants (30 were excluded) were recruited from the First Affiliated Hospital of Zhejiang University School of Medicine, while the remaining 170 participants were from community health service centres. The participants recruited at the hospital will receive standard medical treatment for their health conditions, physical function assessment and will undergo our ultrasound examinations when they are time available. The participants recruited from the community will undergo all study‐related examinations and evaluations following recruitment. Participants first completed demographic and functional assessments, followed by body composition measurements using BIA and DXA, and finally underwent musculoskeletal ultrasound examinations. Community‐dwelling participants completed all the assessments at one time within 1–1.5 h at their scheduled appointment. Hospitalized patients completed physical function, body composition (BIA and DXA), and ultrasound assessments over two consecutive days, scheduled at times accessible to them, without disrupting standardized clinical diagnosis and treatment protocols. All participants underwent ultrasound examination. Additionally, 100 participants were measured a second time by another rater within 3 days to assess inter‐rater reliability, with the assessment results blinded to each rater.

### 2.2 Measurements

#### 2.2.1 Ultrasound Measurements

The M9 ultrasound system with an L20‐5s linear array transducer (Mindray, China) was used to collect muscle ultrasound images. All subjects underwent the ultrasound examinations by a single physician. Of these, 100 participants were measured by a second physician (including a complete re‐landmarking of the measurement sites) at a different time point but within 3 days for inter‐rater reliability assessment. Prior to the study, both physicians completed a two‐week training for muscle ultrasonic examination guided by two experienced sonographers. A total of seven muscle sites and 18 ultrasound parameters were recorded. The Supplementary Table S1 provides a detailed anatomical localization of the respective regions.

**FIGURE 1:**
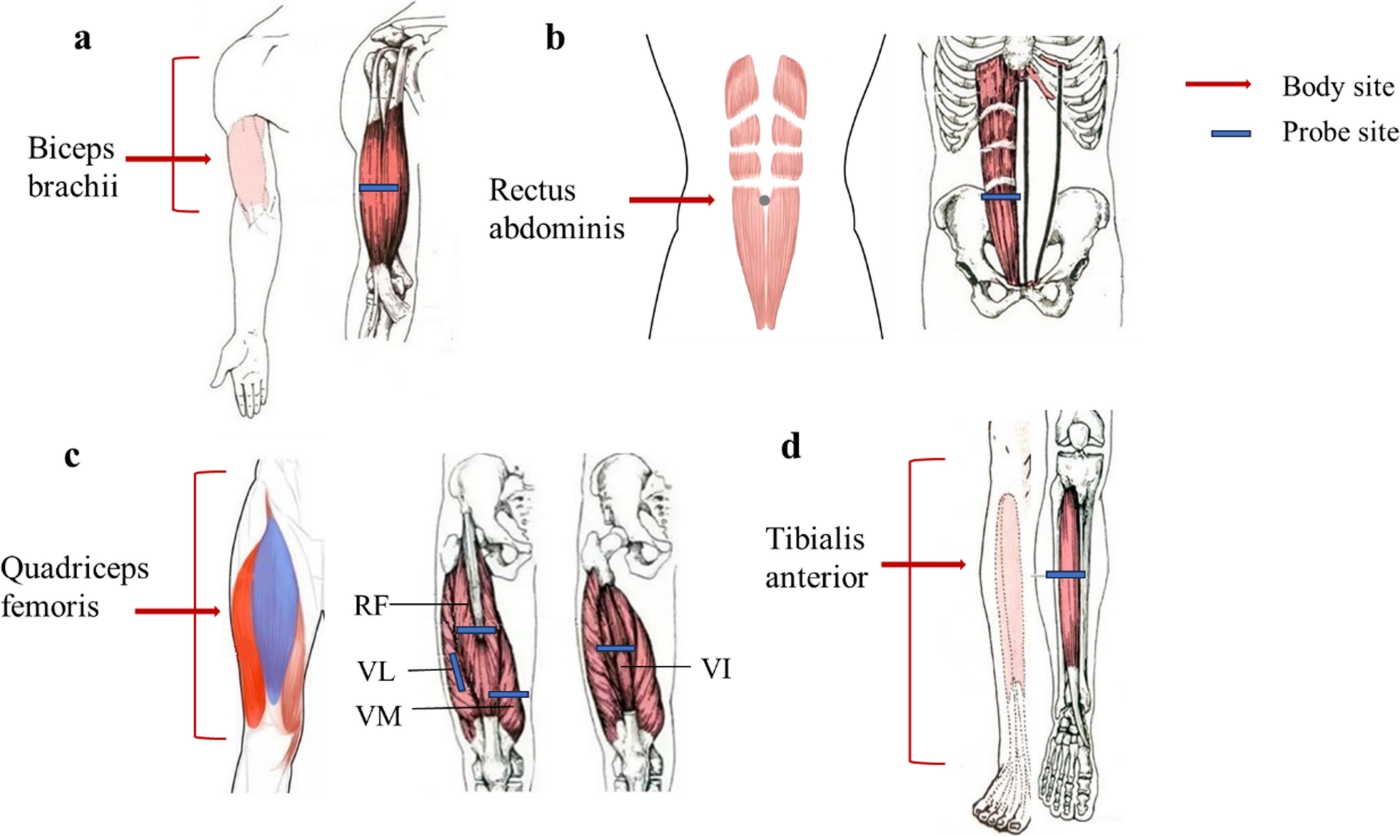
Anatomical landmarks for each muscle. (a) The point of biceps brachii was the thickest part of the dominant arm—about two‐thirds between the acromion and the antecubital crease. (b) The point of rectus abdominis was about 3 cm at the right of the umbilicus. (c) The points of quadriceps femoris, rectus femoris (RF) and vastus intermedius (VI) were the midpoint between the anterior superior spine to the superior border of the patella. The point of vastus lateralis (VL) was the lower two‐thirds between the anterior superior spine to the superior border of the patella. The point of vastus medialis (VM) was about 3–4 cm above the patella. (d) The point of tibialis anterior was the upper one‐quarter between the inferior border of the patella to the lateral malleolus. The red arrow marks the measurement point of each muscle, and the blue mark represents the probe point.

The participants were positioned lying on the bed with their limbs fully relaxed and extended. The supine position allowed the examiner to establish a stable fulcrum, which facilitated more accurate and stable ultrasound imaging. Ultrasound examinations were conducted at the following muscle sites of the right limb, as previously described: biceps brachii [14], rectus abdominis [15], rectus femoris and vastus intermedius [16], vastus lateralis [16], vastus medialis [16] and tibialis anterior [14] (Figure 1 and Supplementary Table S1, Supplementary Figure S1). Ultrasound images of subcutaneous fat thickness (FT) and MT were acquired with the probe lightly placed on the body. For the biceps brachii and rectus abdominis muscles, the pressed MT was also measured with the probe firmly pressed against the body. The CSA was measured at biceps brachii and rectus femoris. The FT was the distance between the skin and the superficial fascia. MT was defined as the distance between deep and superficial aponeurosis. CSA was determined by manually tracing the muscle boundary using a cursor (Supplementary Figure S2). Measurements of MT and FT were derived from a single ultrasound image, while CSA was calculated as the average of two separate image measurements. All images were collected at the transverse section, except for the vastus medialis, which was imaged in the coronal plane (Supplementary Figure S1). Prior to measuring the MT of the biceps brachii, a dynamic scan was performed to visualize the entire cross‐sectional area of the muscle, thereby identifying its lower boundary. This allows for better differentiation of the biceps brachii from the underlying brachialis muscle. When the biceps brachii is compressed, it is more difficult to distinguish it from the underlying brachialis, and dynamic scanning is also hard to practice. Therefore, the distance from the subcutaneous fat to the humerus was selected as the measurement reference (Supplementary Figure S2). Measurements under compression were stopped at maximal resistance, with no further compression possible.

#### 2.2.2 Low Muscle Mass Definition

DXA (Lunar iDXA, GE Healthcare, France) and BIA (BCA‐2A, Tsinghua Tongfang, Beijing, China) were used to measure muscle mass. DXA‐calculated appendicular skeletal muscle mass index (ASMI) was used as the standard for diagnosing low muscle mass, and BIA‐defined muscle mass was a comparison with ultrasound diagnosis. The diagnosis of low muscle mass was based on the revised criteria proposed by the Asian Working Group for Sarcopenia (AWGS) [3]. Low muscle mass was defined as ASMI below 7.0 kg/m^2^ for men and 5.4 kg/m^2^ for women when measured by DXA, or below 7.0 kg/m^2^ for men and 5.7 kg/m^2^ for women when measured by BIA.

In brief, a whole‐body scan was performed using DXA to assess appendicular fat‐free mass, with the height‐squared‐adjusted fat‐free mass recorded as ASMI. For BIA measurements, subjects were instructed to stand upright on foot electrodes and hold hand electrodes according to the manufacturer's instructions. The procedures were as previously described [17].

#### 2.2.3 Grip Strength

Grip strength was measured using a JAMAR plus+ hand dynamometer, which can show an isometric grip strength ranging from 0 to 200 lbs (90 kg). Participants were instructed to grip the device forcefully with their dominant hand, while keeping their arm at their side and elbow flexed at a 90° angle. The dynamometer was configured at the second handle position by default, and the grip width was individually adjusted to match the participant's hand size when required [18]. The maximum value from three attempts was recorded for the dominant hand [19]. The interval time between grip tests was 30 s.

#### 2.2.4 Gait Speed

A 4‐m test was conducted to assess the gait speed. Participants were guided to walk at their usual pace over a 6‐m course in a designated area, with the middle 4 m used to calculate gait speed [20].

#### 2.2.5 Activities of Daily Living

The Barthel index of activities of daily living (ADL) was evaluated as the ability of activities. ADL includes 10 components (feeding, bathing, dressing, grooming, toileting, bowel control, bladder control, ambulation, chair transfer and stair climbing) [21]. The grade of each item was defined as 15, 10, 5 or 0 points. The final score (ranging from 0 to 100) was the sum of the grades of all items. A score of 100 indicated good function, while scores between 75 and 95 indicated mild impairment, 50 to 70 indicated moderate impairment and scores ≤ 45 indicated severe impairment.

#### 2.2.6 Physical Performance

Physical performance was assessed by the Short Physical Performance Battery (SPPB). SPPB consisted of gait speed, chair stand test and balance [21]. Each item was scored from 0 to 4, with higher scores indicating better performance. The scores from these three items were added together to produce a total score ranging from 0 to 12. A total score of 0 to 6 indicated poor performance, 7 to 9 indicated moderate performance and 10 to 12 indicated good performance [22].

#### 2.2.7 Frailty Definition

The frailty was assessed based on the Fried phenotype model [23]. According to this model, the frailty phenotype is defined by five components: unintentional weight loss, weakness, low grip strength, slow gait speed and low physical activity. Participants meeting three or more criteria were classified as frail, while those meeting one or two criteria were classified as pre‐frail [23].

#### 2.2.8 Other Characteristics

Participants' age, height, weight and BMI were recorded. Height and weight were measured using standardized scales (Seca 764 digital scale, Germany) while participants stood upright. BMI was calculated by dividing weight (kilograms) by height (meters squared). Additionally, information on education level and living status was collected through interviews.

### 2.3 Statistical Analysis

Data were presented as means ± SEM for normal distribution, while medians and interquartile ranges (IQRs) for non‐normal distribution. The Spearman test was performed to examine the bivariate correlation. Receiver operating characteristic (ROC) curve analysis and 10‐fold cross validation were conducted to evaluate the prediction performance of ultrasound parameters for low muscle mass. The Youden index was employed to determine optimal cut‐offs, while sensitivity, specificity and accuracy were calculated to assess diagnostic reliability.

Ordinal logistic regression models were utilized to assess the association between low muscle mass defined by ultrasound parameters and health outcomes including ability of activities, physical performance and frailty status, as compared with DXA and BIA defined low muscle mass. These outcomes were defined as ordered categorical variables. The ability of activities was categorized as good function (a score of 100), mild impairment (scores of 75 to 95), moderate impairment (scores of 50 to 70) and severe impairment (scores ≤ 45). Physical performance was classified into good (score of 10 to 12), moderate (scores of 7 to 9) and poor performance (scores of 0 to 6). Frailty status was defined as normal (0 items), pre‐frail (1 or 2 items) and frailty (3 to 5 items). ICC was performed to evaluate inter‐rater reliability between two ultrasound examiners. *p* < 0.05 was considered a significant difference. IBM SPSS Statistics 24.0 and Stata 18.0 were used for all data analyses.

## 3 Results

### 3.1 Participant Characteristics and Ultrasound Parameters

A total of 1011 subjects were included in data analysis. All participants completed ultrasound assessments, and 816 underwent muscle mass evaluation using DXA. Table 1 summarized the participants' characteristics. The median age was 75 years for males and 74 years for females. Males had greater MT and CSA compared with females, while females had greater FT.

**TABLE 1:** Characteristics of the study population.

Characteristic	Male *n* = 543	Female *n* = 468
Age, year, median (IQR)	75 (69–82)	74 (68–80)
Height, cm, median (IQR)	165 (161–170)	154 (150–158)
Weight, kg, median (IQR)	62.1 (55.0–70.0)	53.6 (47.4–60.0)
BMI, kg/m^2^, median (IQR)	22.9 (20.4–25.3)	22.6 (20.1–25.2)
Education level, *n* (%)		
Primary school or uneducated	157 (33.7)	200 (50.0)
Junior school	147 (31.5)	105 (26.3)
High school	81 (17.4)	69 (17.3)
University bachelor's degree or postgraduate degree	81 (17.4)	26 (6.4)
Living status, *n* (%)		
Living alone	40 (9.2)	53 (13.8)
Not living alone	397 (90.8)	330 (86.2)
Physical performance, *n* (%)		
Good performance	247 (57.6)	212 (56.2)
Moderate performance	120 (28.0)	98 (26.0)
Poor performance	62 (14.5)	67 (17.8)
Ability of activities, *n* (%)		
Good function	360 (77.4)	321 (80.9)
Mild impairment	70 (15.1)	51 (12.8)
Moderate impairment	21 (4.5)	20 (5.0)
Severe impairment	14 (3.0)	5 (1.3)
Fried‐Frailty, *n* (%)		
Good	182 (39.3)	165 (41.8)
Pre‐frail	171 (36.9)	143 (36.2)
Frail	110 (23.8)	87 (22.0)
DXA, kg/m^2^, median (IQR)	6.7 (6.0–7.3)	5.7 (53–6.3)
BIA, kg/m^2^, median (IQR)	7.3 (6.5–8.1)	5.6 (4.8–6.5)
Grip strength, kg, median (IQR)	30 (23–35)	20 (16–24)
Gait speed, m/s, median (IQR)	1.1 (0.8–1.3)	1.0 (0.8–1.3)
Ultrasound parameters, median (IQR)		
Biceps brachii FT, cm	0.22 (0.14–0.33)	0.44 (0.30–0.57)
Biceps brachii MT, cm	2.06 (1.86–2.27)	1.63 (1.47–1.77)
Pressed‐biceps brachii MT, cm	1.80 (1.51–2.04)	1.37 (1.16–1.60)
Biceps brachii CSA, cm^2^	6.86 (5.67–7.95)	4.53 (3.88–5.27)
Rectus abdominis FT, cm	1.60 (1.13–2.05)	2.37 (1.81–2.93)
Rectus abdominis MT, cm	0.85 (0.72–0.96)	0.64 (0.53–0.74)
Pressed‐rectus abdominis MT, cm	0.67 (0.58–0.77)	0.52 (0.44–0.60)
Rectus femoris FT, cm	0.51 (0.35–0.68)	0.87 (0.64–1.13)
Rectus femoris MT, cm	1.49 (1.25–1.68)	1.34 (1.14–1.52)
Vastus intermedius MT, cm	1.19 (0.91–1.49)	1.06 (0.83–1.32)
Quadriceps femoris MT, cm	1.31 (1.05–1.58)	1.10 (0.88–1.36)
Rectus femoris CSA, cm^2^	4.67 (3.91–5.54)	3.69 (3.16–4.42)
Vastus lateralis FT, cm	0.19 (0.11–0.30)	0.53 (0.32–0.74)
Vastus lateralis MT, cm	1.52 (1.26–1.80)	1.43 (1.13–1.65)
Vastus medialis FT, cm	0.38 (0.25–0.50)	0.69 (0.51–0.90)
Vastus medialis MT, cm	2.98 (2.65–3.31)	2.66 (2.45–2.94)
Tibialis anterior FT, cm	0.07 (0.05–0.12)	0.16 (0.09–0.24)
Tibialis anterior MT, cm	2.61 (2.36–2.87)	2.35 (2.15–2.55)

Abbreviations: ADL, Activities of daily living; BIA, muscle mass measured by BIA; CSA, muscle cross‐sectional area; DXA, muscle mass measured by DXA; FT, fat thickness; MT, muscle thickness.

Table 2 showed the differences of ultrasound parameters between low muscle mass group and normal muscle mass group defined by DXA. The MT values were significantly lower in low muscle mass group than normal muscle mass group in both males and females. While no significant differences were observed in the FT values of vastus lateralis and tibialis anterior between the two groups.

**TABLE 2:** Participants' ultrasound parameters for DXA‐defined low muscle mass.

Characteristic	Men (*n* = 456)			Women (*n* = 360)		
	Low muscle mass *n* = 283	Normal muscle mass *n* = 173	*p*	Low muscle mass *n* = 111	Normal muscle mass *n* = 249	*P*
Age, year, median (IQR)	77 (70–84)	72 (66–77)	***< 0.001***	74 (68, 81)	72 (67, 77)	***0.018***
Height, cm, median (IQR)	165 (160–169)	167 (162–171)	***0.008***	153 (149–157)	155 (150–159)	***0.010***
Weight, kg, median (IQR)	59.4 (52.9–64.3)	70.4 (65.0–77.9)	***< 0.001***	47.0 (41.9–51.8)	58.0 (53.0–64.4)	***< 0.001***
BMI, kg/m^2^, median (IQR)	21.7 (19.7–23.9)	25.5 (23.6–27.4)	***< 0.001***	20.1 (18.1, 22.0)	24.3 (22.4, 26.5)	***< 0.001***
Biceps brachii FT, cm	0.21 (0.13–0.32)	0.25 (0.17–0.34)	***0.007***	0.37 (0.27–0.50)	0.49 (0.37–0.64)	***< 0.001***
Biceps brachii MT, cm	1.95 (1.77–2.13)	2.27 (2.10–2.42)	***< 0.001***	1.52 (1.39–1.64)	1.70 (1.56–1.85)	***< 0.001***
Pressed‐biceps brachii MT, cm	1.65 (1.41–1.91)	1.98 (1.76–2.27)	***< 0.001***	1.20 (0.98–1.41)	1.48 (1.24–1.65)	***< 0.001***
Biceps brachii CSA, cm^2^	6.21 (5.38–7.23)	7.97 (7.20–8.98)	***< 0.001***	3.97 (3.57–4.49)	4.90 (4.33–5.66)	***< 0.001***
Rectus abdominis FT, cm	1.45 (1.02–1.95)	1.79 (1.43–2.27)	***< 0.001***	2.04 (1.40–2.61)	2.51 (1.98–3.11)	***< 0.001***
Rectus abdominis MT, cm	0.82 (0.68–0.92)	0.93 (0.81–1.06)	***< 0.001***	0.60 (0.48–0.69)	0.67 (0.56–0.75)	***< 0.001***
Pressed‐rectus abdominis MT, cm	0.63 (0.54–0.72)	0.74 (0.65–0.84)	***< 0.001***	0.49 (0.41–0.58)	0.54 (0.47–0.61)	***0.001***
Rectus femoris FT, cm	0.48 (0.33–0.66)	0.56 (0.38–0.73)	0.023	0.80 (0.54–1.06)	0.94 (0.72–1.20)	0.004
Rectus femoris MT, cm	1.40 (1.18–1.60)	1.67 (1.49–1.81)	***< 0.001***	1.18 (1.02–1.36)	1.41 (1.25–1.57)	***< 0.001***
Vastus intermedius MT, cm	1.01 (0.85–1.34)	1.44 (1.20–1.70)	***< 0.001***	0.87 (0.69–1.10)	1.19 (0.95–1.42)	***< 0.001***
Quadriceps femoris MT, cm	1.19 (0.97–1.42)	1.57 (1.32–1.86)	***< 0.001***	0.89 (0.68–1.10)	1.22 (1.01–1.44)	***< 0.001***
Rectus femoris CSA, cm^2^	4.25 (3.64–5.03)	5.39 (4.70–6.05)	***< 0.001***	3.16 (2.86–3.86)	3.93 (3.42–4.65)	***< 0.001***
Vastus lateralis FT, cm	0.18 (0.10–0.28)	0.20 (0.12–0.32)	0.113	0.53 (0.29–0.74)	0.56 (0.34–0.78)	0.083
Vastus lateralis MT, cm	1.38 (1.17–1.62)	1.74 (1.52–2.00)	***< 0.001***	1.16 (0.92–1.46)	1.51 (1.26–1.72)	***< 0.001***
Vastus medialis FT, cm	0.39 (0.25–0.50)	0.37 (0.24–0.51)	0.858	0.65 (0.48–0.78)	0.74 (0.55–0.94)	***< 0.001***
Vastus medialis MT, cm	2.84 (2.56–3.19)	3.24 (2.89–3.48)	***< 0.001***	2.54 (2.31–2.74)	2.81 (2.57–3.04)	***< 0.001***
Tibialis anterior FT, cm	0.07 (0.05–0.12)	0.07 (0.05–0.11)	0.744	0.16 (0.07–0.24)	0.16 (0.10–0.25)	0.075
Tibialis anterior MT, cm	2.49 (2.24–2.69)	2.86 (2.66–3.06)	***< 0.001***	2.11 (1.90–2.30)	2.46 (2.30–2.63)	***< 0.001***

*Note:* Data are represented as median (25–75th percentiles) or *n* (%). Categorical variables were analysed by chi‐square test, while other continuous data were analysed by Mann–Whitney U test. Bold and italic formatting indicates statistical significance *p* <0.05.

Abbreviations: CSA, muscle cross‐sectional area; FT, fat thickness; MT, muscle thickness.

### 3.2 Bivariate Correlations Between Ultrasound Muscle Parameters and Other Indicators

Correlations between ultrasound parameters and age, BMI, DXA‐assessed ASMI, BIA‐assessed ASMI, grip strength and gait speed were presented as supplementary Figure S3. The negative correlations were observed between age and MT values or CSA values in both males and females, as well as muscle mass defined by DXA or BIA. Moderate positive correlations were found between ultrasound muscle MT values or CSA values and BMI, DXA‐assessed ASMI, BIA‐assessed ASMI. The strongest correlations observed between ultrasound parameters and DXA‐assessed ASMI were biceps brachii CSA in males with Spearman's r = 0.697 and tibialis anterior MT in females with r = 0.641, while the correlations between gait speed and ultrasound parameters were relatively poor.

### 3.3 Prediction, Diagnostic Performance and Cut‐Off Values of Ultrasound Parameters for Low Muscle Mass

**FIGURE 2:**
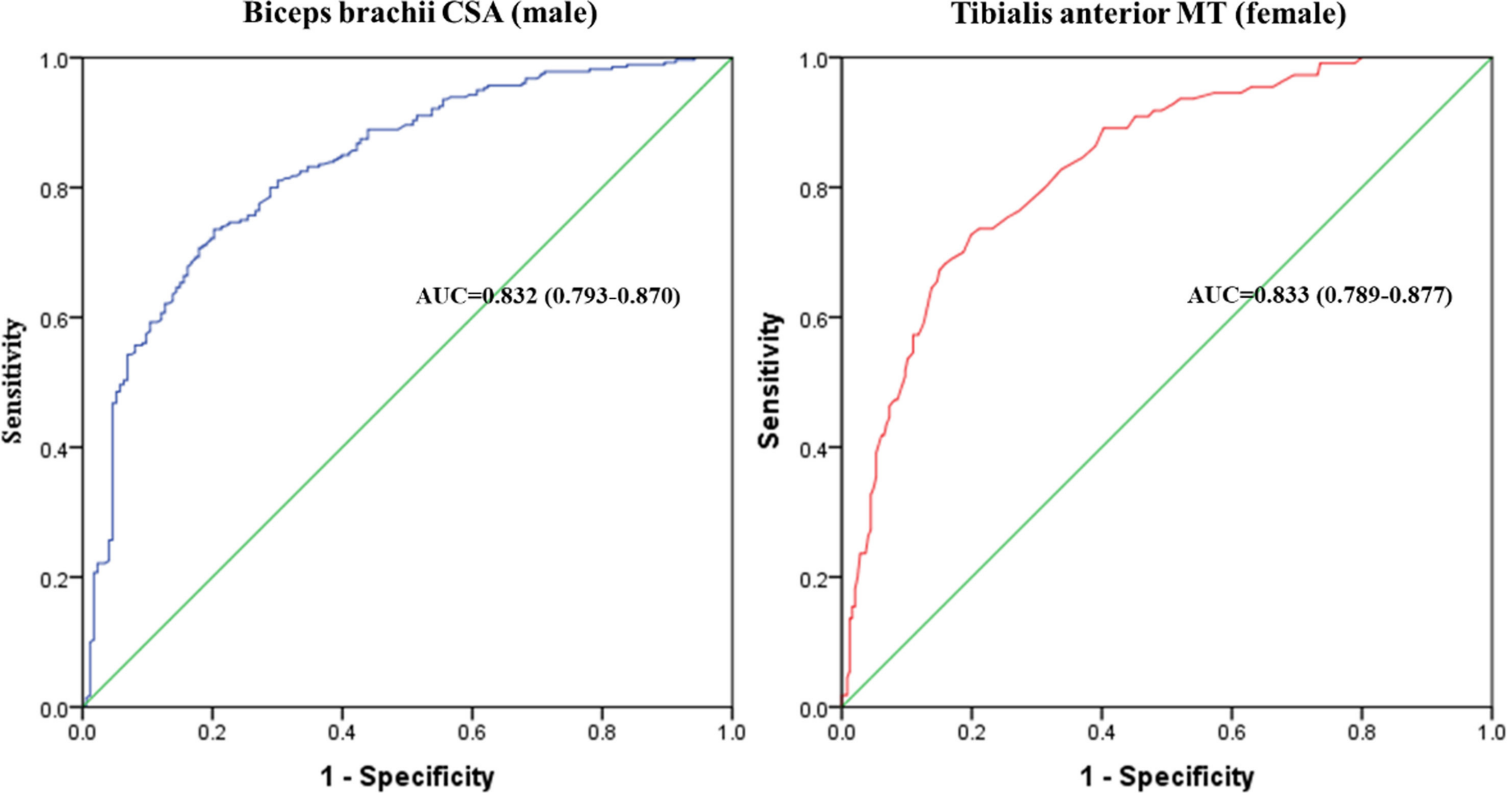
The largest ROC curve of ultrasound parameters to predict low muscle mass defined by DXA in male and female. The largest ROC curve was biceps brachii CSA in male and tibialis anterior MT in female, respectively.

Table 3 presented the area under the curve (AUC), positive predictive value (PPV), negative predictive value (NPV) and accuracy of ultrasound parameters in predicting low muscle mass defined by DXA. The biceps brachii CSA (AUC = 0.832, 95% CI: 0.793–0.870) in male and tibialis anterior MT (0.833, 95% CI: 0.789–0.877) in female demonstrated superior predictive power for muscle mass compared with other assessed parameters (Table 3 and Figure 2). These findings were validated through 10‐fold cross‐validation. We also calculated the AUC values for ultrasound parameters adjusted for height, height squared, weight, and BMI (by dividing the ultrasound parameters by these variables, respectively) (Supplementary Table S3). However, the resulting AUC values were lower than those of the unadjusted ultrasound parameters.

**TABLE 3:** The AUC (95% CI), PPV, NPV and accuracy of ultrasound parameter prediction models for DXA‐defined low muscle mass and internal validation.

Measurements	Univariate prediction model				10‐Fold cross validation			
	AUC (95% CI)	PPV (%)	NPV (%)	Accuracy (%)	AUC	PPV (%)	NPV (%)	Accuracy (%)
Men								
Biceps brachii CSA, cm^2^	0.832 (0.793–0.870)***	77.9%	70.7%	75.5%	0.831	77.6%	70.5%	75.3%
Biceps brachii MT, cm	0.809 (0.768–0.849)***	76.2%	66.5%	72.9%	0.807	76.2%	66.5%	72.9%
Tibialis anterior MT, cm	0.803 (0.761–0.844)***	77.3%	68.5%	74.4%	0.799	77.1%	68.7%	74.4%
Quadriceps femoris MT, cm	0.783 (0.740–0.826)***	73.9%	69.9%	72.8%	0.780	74.1%	68.8%	72.6%
Rectus femoris CSA, cm^2^	0.775 (0.732–0.817)***	73.0%	64.0%	70.3%	0.771	72.8%	63.7%	70.1%
Vastus intermedius MT, cm	0.766 (0.722–0.811)***	72.8%	63.0%	69.8%	0.765	72.6%	63.2%	69.8%
Vastus lateralis MT, cm	0.761 (0.717–0.806)***	72.5%	63.4%	69.9%	0.761	71.9%	62.5%	69.2%
Pressed‐biceps brachii MT, cm	0.754 (0.708–0.800)***	71.8%	65.1%	69.9%	0.752	71.5%	64.3%	69.4%
Rectus femoris MT, cm	0.750 (0.705–0.795)***	73.3%	64.9%	70.8%	0.749	73.2%	65.2%	70.8%
Pressed‐rectus abdominis MT, cm	0.720 (0.672–0.769)***	69.9%	63.6%	68.4%	0.719	69.4%	62.4%	67.7%
Vastus medialis MT, cm	0.713 (0.665–0.762)***	70.0%	60.2%	67.5%	0.710	70.1%	60.7%	67.7%
Rectus abdominis MT, cm	0.703 (0.654–0.752)***	69.8%	67.0%	69.2%	0.701	69.6%	65.0%	68.6%
Women								
Tibialis anterior MT, cm	0.833 (0.789–0.877)***	70.0%	80.4%	78.1%	0.831	70.0%	80.4%	78.1%
Biceps brachii CSA, cm^2^	0.782 (0.733–0.831)***	59.0%	76.7%	72.8%	0.779	59.0%	76.7%	72.8%
Quadriceps femoris MT, cm	0.760 (0.705–0.814)***	64.2%	77.2%	74.7%	0.758	63.2%	77.1%	74.4%
Rectus femoris CSA, cm^2^	0.756 (0.701–0.810)***	74.2%	79.2%	78.3%	0.755	74.2%	79.2%	78.3%
Vastus intermedius MT, cm	0.744 (0.690–0.798)***	62.1%	76.2%	73.6%	0.742	62.9%	75.9%	73.6%
Rectus femoris MT, cm	0.742 (0.686–0.798)***	69.2%	75.7%	74.7%	0.739	69.2%	75.7%	74.7%
Biceps brachii MT, cm	0.738 (0.685–0.791)***	53.9%	73.0%	70.2%	0.736	54.0%	72.8%	70.2%
Pressed‐biceps brachii MT, cm	0.727 (0.670–0.783)***	60.0%	74.8%	72.3%	0.723	60.7%	75.0%	72.6%
Vastus lateralis MT, cm	0.724 (0.666–0.782)***	61.8%	74.8%	72.8%	0.719	60.0%	75.0%	72.5%
Vastus medialis MT, cm	0.713 (0.655–0.771)***	63.0%	73.9%	72.5%	0.708	61.7%	73.8%	72.2%
Rectus abdominis MT, cm	0.627 (0.562–0.691)***	83.3%	70.3%	70.5%	0.624	83.3%	70.3%	70.5%
Pressed‐rectus abdominis MT, cm	0.611 (0.546–0.676)***	50.0%	69.5%	69.3%	0.601	33.3%	69.3%	69.0%

*Note:* Univariate analysis of ultrasound parameters was conducted to predict DXA‐defined low muscle mass, and 10‐fold cross‐validation was employed for internal validation. Data are represented as median (25–75th percentiles).

Abbreviations: AUC, area under the curve; CSA, muscle cross‐sectional area; FT, fat thickness; MT, muscle thickness; NPV, negative predictive value; PPV, positive predictive value.

*** *p* < 0.001.

Table 4 showed the cut‐offs, sensitivity, specificity and accuracy of ultrasound parameters for diagnosing low muscle mass. When DXA‐defined low muscle mass was used as the standard, the optimal cut‐offs for ultrasound measurements were biceps brachii CSA < 7.1 cm^2^ for males and tibialis anterior MT < 2.3 cm for females according to ROC and Youden index. The sensitivity, specificity and accuracy were 73.9%, 78.6% and 75.7% in biceps brachii CSA in males, and 73.6%, 76.8% and 75.8% in tibialis anterior MT in females. The diagnosis performances of ultrasound were comparable with BIA, of which had sensitivity, specificity, and accuracy of 62.6%, 80.2%, and 71.5% respectively.

**TABLE 4:** Diagnostic performance of ultrasound parameters for diagnosing low muscle mass based on DXA diagnostic criteria and associations between ultrasound defined low muscle mass and physical performance, ability of activities and Frailty status.

Ultrasound parameters	Cut‐offs	Sensitivity (%)	Specificity (%)	Accuracy (%)	Odds ratio		
					Physical performance	Ability of activities	Frailty status
Men							
Biceps brachii CSA, cm^2^	7.1	73.9%	78.6%	75.7%	2.511 (1.703–3.701)	3.002 (1.861–4.840)	3.193 (2.234–4.562)
Tibialis anterior MT, cm	2.7	75.2%	70.2%	73.3%	2.051 (1.388–3.031)	2.620 (1.601–4.289)	3.295 (2.291–4.739)
Rectus femoris CSA, cm^2^	5.0	73.8%	68.2%	71.6%	1.527 (1.044–2.235)	1.784 (1.131–2.814)	2.027 (1.430–2.873)
Vastus lateralis MT, cm	1.5	62.9%	79.7%	69.2%	1.973 (1.354–2.875)	1.744 (1.126–2.703)	2.766 (1.950–3.925)
Pressed‐biceps brachii MT, cm	1.9	73.3%	62.2%	69.0%	2.396 (1.604–3.581)	2.576 (1.548–4.288)	2.870 (1.998–4.124)
Quadriceps femoris MT, cm	1.3	61.9%	80.1%	68.8%	2.031 (1.392–2.965)	2.092 (1.330–3.291)	3.178 (2.227–4.536)
Biceps brachii MT, cm	2.0	58.0%	86.1%	68.7%	2.243 (1.533–3.282)	2.623 (1.687–4.079)	2.877 (2.015–4.109)
Vastus intermedius MT, cm	1.1	59.1%	83.2%	68.3%	2.189 (1.501–3.194)	2.635 (1.685–4.120)	3.697 (2.579–5.299)
Rectus femoris MT, cm	1.5	64.7%	73.4%	68.0%	1.388 (0.957–2.012)	2.254 (1.443–3.522)	2.464 (1.742–3.487)
Vastus medialis MT, cm	3.1	70.3%	62.8%	67.5%	1.797 (1.227–2.632)	2.620 (1.626–4.223)	1.937 (1.371–2.737)
Rectus abdominis MT, cm	0.9	69.9%	60.7%	66.4%	1.726 (1.170–2.546)	1.775 (1.112–2.832)	2.257 (1.585–3.215)
Pressed‐rectus abdominis MT, cm	0.6	39.7%	87.8%	58.1%	1.611 (1.071–2.423)	1.540 (0.961–2.467)	2.835 (1.928–4.169)
Women							
Rectus femoris CSA, cm^2^	3.2	51.4%	89.0%	77.5%	1.403 (0.892–2.207)	1.427 (0.829–2.459)	1.802 (1.180–2.752)
Tibialis anterior MT, cm	2.3	73.6%	76.8%	75.8%	2.033 (1.359–3.040)	2.104 (1.272–3.482)	2.174 (1.482–3.191)
Biceps brachii CSA, cm^2^	4.4	70.3%	73.3%	72.3%	1.626 (1.093–2.420)	1.794 (1.084–2.971)	2.008 (1.378–2.925)
Vastus lateralis MT, cm	1.2	51.8%	80.1%	71.3%	1.797 (1.175–2.750)	1.540 (0.912–2.599)	2.229 (1.475–3.368)
Rectus femoris MT, cm	1.3	66.4%	71.1%	69.7%	1.904 (1.277–2.838)	1.377 (0.836–2.268)	2.226 (1.525–3.250)
Biceps brachii MT, cm	1.6	67.6%	67.7%	67.7%	1.678 (1.131–2.491)	1.566 (0.951–2.581)	1.669 (1.150–2.421)
Vastus medialis MT, cm	2.6	61.8%	70.3%	67.7%	1.216 (0.817–1.812)	1.861 (1.124–3.082)	1.647 (1.130–2.401)
Quadriceps femoris MT, cm	1.1	75.0%	62.7%	66.5%	1.705 (1.142–2.544)	1.843 (1.099–3.092)	1.685 (1.158–2.451)
Pressed‐biceps brachii MT, cm	1.4	73.9%	58.9%	63.6%	1.678 (1.125–2.502)	1.364 (0.821–2.264)	1.664 (1.146–2.416)
Pressed‐rectus abdominis MT, cm	0.5	51.4%	65.0%	60.8%	0.934 (0.626–1.392)	0.604 (0.353–1.033)	0.936 (0.642–1.365)
Vastus intermedius MT, cm	1.2	86.4%	49.2%	60.7%	1.877 (1.227–2.869)	2.456 (1.356–4.448)	2.035 (1.371–3.021)
Rectus abdominis MT, cm	0.7	77.1%	39.3%	50.8%	0.543 (0.360–0.820)	0.491 (0.296–0.817)	0.633 (0.428–0.936)

*Note:* ROC curve analysis and Youden index calculations were performed to determine the optimal cut‐offs for ultrasound parameters in diagnosing low muscle mass based on the DXA diagnostic criteria of low muscle mass. The sensitivity, specificity and accuracy were calculated to evaluate the diagnostic performance of the ultrasound parameters in identifying low muscle mass. Using DXA as the reference standard, the sensitivity, specificity and accuracy of BIA were 62.6%, 80.2% and 71.5% respectively. Ordinal logistic regression was analysed to assess the associations between ultrasound‐defined low muscle mass and health outcomes such as the physical performance, ability of activities and Frailty status. The physical performance was assessed by the Short Physical Performance Battery, which were categorized as good (score of 10 to 12), moderate (scores of 7 to 9) and poor performance (scores of 0 to 6). Ability of activities was calculated by the Barthel index of activities of daily living and defined as good function (a score of 100), mild impairment (scores of 75 to 95), moderate impairment (scores of 50 to 70) and severe impairment (scores ≤ 45). The frailty was defined by Fried phenotype model and classified into normal (0 item), pre‐frail (1 or 2 items) and frailty (3 to 5 items).

Abbreviations: AUC, area under the curve; CSA, muscle cross‐sectional area; FT, fat thickness; MT, muscle thickness.

**FIGURE 3:**
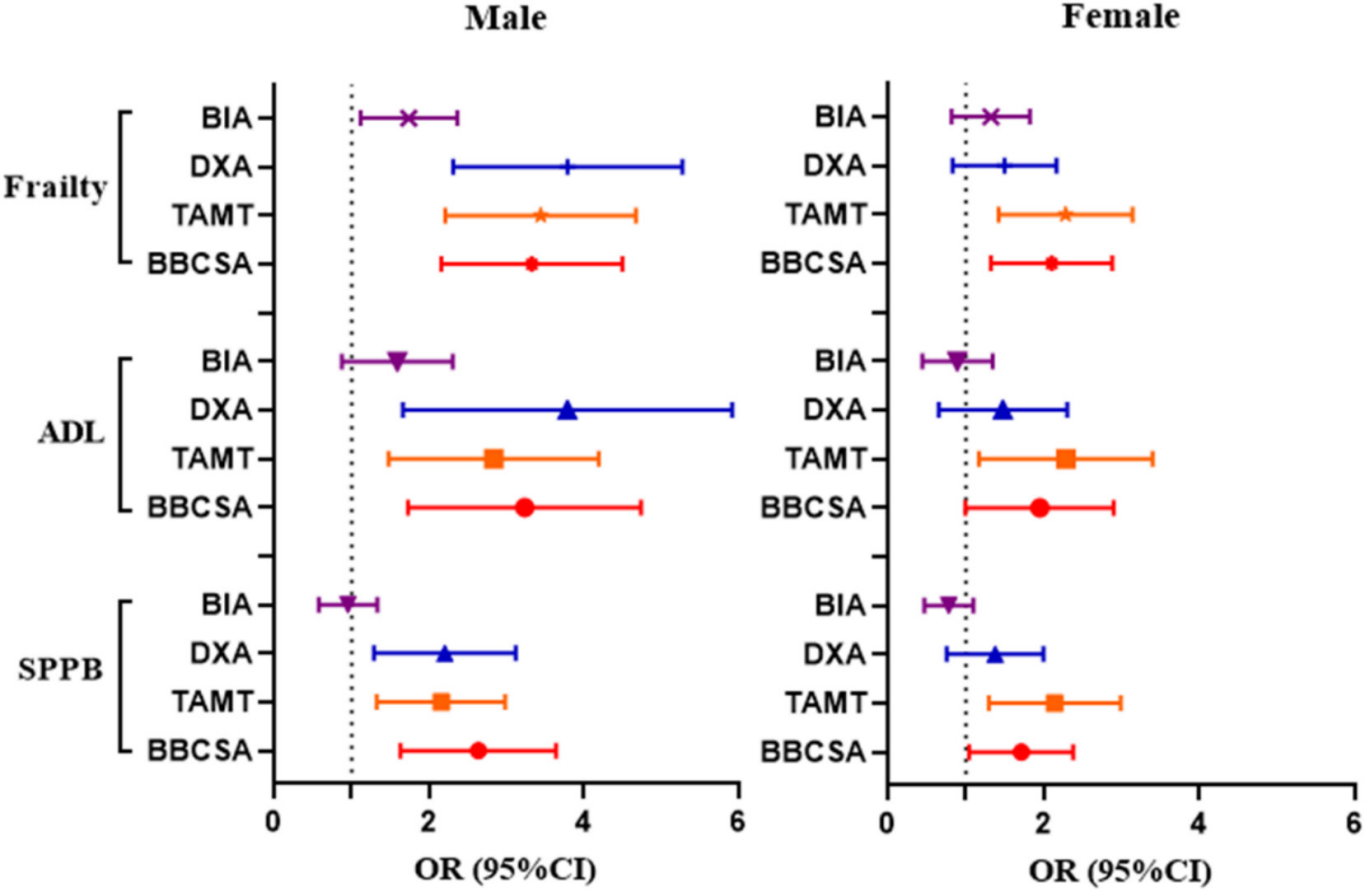
Associations between low muscle mass defined by DXA, BIA and ultrasound biceps brachii CSA, and tibialis anterior MT with health outcomes. Ordinal logistic regression models were utilized to assess the association between muscle mass (binary variable) and health outcomes. Frailty status was defined as normal (0 item), prefrail (1 or 2 items) and frailty (3 to 5 items). The ADL was categorized as good function (a score of 100), mild impairment (scores of 75 to 95), moderate impairment (scores of 50 to 70) and severe impairment (scores ≤ 45). The SPPB was classified into good (score of 10 to 12), moderate (scores of 7 to 9) and poor performance (scores of 0 to 6). ADL: activities of daily living; BBCSA: muscle mass defined by biceps brachii muscle cross‐section area; BIA: muscle mass defined by bioelectrical impedance analysis; CI: confidence interval; DXA: muscle mass defined by dual‐energy X‐ray absorptiometry; OR: odds ratio; SPPB: Short Physical Performance Battery; TAMT: muscle mass defined by tibialis anterior muscle thickness.

The muscle mass, as delineated by ultrasound parameters, demonstrated a significant association with physical performance, ability of activities and frailty status through ordinal logistic regression analysis (Table 4 and Figure 3). Among male participants, the biceps brachii CSA exhibited the highest odds ratios (ORs), specifically 2.511 (95% CI: 1.703–3.701) for physical performance, 3.002 (95% CI: 1.861–4.840) for ability of activities and 3.193 (95% CI: 2.234–4.562) for frailty status. In contrast, for female participants, the tibialis anterior MT showed the greatest impact, with ORs of 2.033 (95% CI: 1.359–3.040) for physical performance, 2.104 (95% CI: 1.272–3.482) for ability of activities and 2.174 (95% CI: 1.482–3.191) for frailty status, compared with other ultrasound parameters (Table 4). It is noteworthy that in males, the tibialis anterior MT, and in females, the biceps brachii CSA also presented relatively elevated OR values.

Interestingly, the ORs of both biceps brachii CSA and tibialis anterior MT surpassed those of BIA when assessing low muscle mass associated with physical performance, functional ability and frailty status individually (Figure 3). Furthermore, among female subjects, the OR values for biceps brachii CSA and tibialis anterior MT were greater than those obtained from DXA. Additionally, concerning physical performance in males, the OR value for biceps brachii CSA was also found to be higher than that of DXA (Figure 3). These findings highlight the potential superiority of ultrasound parameters over traditional methods in evaluating health outcomes.

### 3.4 Inter‐Rater Reliability of Ultrasound Parameters

ICC analysis showed excellent inter‐rater reliability for biceps brachii CSA (ICC = 0.869, 95% CI: 0.650–0.937) and quadriceps femoris MT (ICC = 0.834, 95% CI: 0.762–0.886). Good reliability was observed for biceps brachii MT (ICC = 0.795, 95% CI: 0.709–0.858), rectus abdominis MT (ICC = 0.790, 95% CI: 0.675–0.863), vastus intermedius MT (ICC = 0.732, 95% CI: 0.625–0.812) and tibialis anterior MT (ICC = 0.730, 95% CI: 0.622–0.810) (Table 5).

**TABLE 5:** Inter‐rater reliability of ultrasound parameters.

Measurements	Total (*n* = 100)			
	Rater 1	Rater 2	ICC (95% CI)	*p*
Biceps brachii CSA, cm^2^	5.72 (4.68–7.26)	5.01 (4.18–6.54)	0.869 (0.650–0.937)	< 0.001
Quadriceps femoris MT, cm	1.07 (0.84–1.33)	1.06 (0.83–1.35)	0.834 (0.762–0.886)	< 0.001
Pressed‐rectus abdominis MT, cm	0.62 (0.52–0.68)	0.58 (0.49–0.67)	0.831 (0.741–0.889)	< 0.001
Biceps brachii MT, cm	1.80 (1.50–2.07)	1.75 (1.56–2.04)	0.795 (0.709–0.858)	< 0.001
Rectus abdominis MT, cm	0.78 (0.65–0.91)	0.72 (0.62–0.85)	0.790 (0.675–0.863)	< 0.001
Vastus intermedius MT, cm	0.98 (0.82–1.25)	0.99 (0.79–1.29)	0.732 (0.625–0.812)	< 0.001
Tibialis anterior MT, cm	2.36 (2.06–2.57)	2.44 (2.12–2.67)	0.730 (0.622–0.810)	< 0.001
Pressed‐biceps brachii MT, cm	1.29 (1.10–1.53)	1.44 (1.24–1.73)	0.659 (0.382–0.801)	< 0.001
Rectus femoris CSA, cm^2^	3.80 (3.19–4.53)	3.92 (3.20–4.67)	0.626 (0.491–0.732)	< 0.001
Vastus lateralis MT, cm	1.64 (1.39–1.88)	1.46 (1.13–1.70)	0.462 (0.203–0.641)	< 0.001
Rectus femoris MT, cm	1.12 (0.89–1.28)	1.34 (1.13–1.52)	0.433 (0.149–0.628)	< 0.001
Vastus medialis MT, cm	3.30 (2.94–3.67)	2.79 (2.56–3.14)	0.408 (0.040–0.641)	< 0.001

*Note:* Intraclass correlation coefficient was conducted to evaluate inter‐rater reliability. Data are represented as median (25–75th percentiles).

Abbreviations: CSA, muscle cross‐sectional area; FT, fat thickness; ICC, intraclass correlation coefficient; MT, muscle thickness.

## 4 Discussion

By including a large sample of older Chinese adults, we assessed several ultrasound muscle parameters to determine the optimal sites and cut‐offs for diagnosing low muscle mass by sex. Our study revealed significantly positive associations between many ultrasound parameters and muscle mass, and there were sex differences in these associations. Of these parameters, the biceps brachii CSA < 7.1 cm^2^ in males and tibialis anterior MT < 2.3 cm in females were superior indicators for diagnosing low muscle mass. Importantly, the low muscle mass defined by these two ultrasound thresholds was significantly associated with poor physical functions, with the effect size being higher than DXA or BIA defined low muscle mass. Moreover, there was good inter‐rater reliability of biceps brachii CSA and tibialis anterior MT. Our findings suggested that ultrasound measurements were more closely associated with physical performance than those assessed by DXA or BIA, thus might serve as a preferable tool for assessing low muscle mass in older Chinese adults.

Our study identified the values of specific muscle parameters in diagnosing sarcopenia, highlighting notable differences between sex. For males, the AUC for the biceps brachii CSA was found to be superior compared with other parameters when evaluating muscle mass. For females, the AUC for the tibialis anterior MT stood out as being higher than other evaluated parameters. This finding confirms our previous research identifying the biceps brachii CSA as a promising indicator for predicting sarcopenia [24], and indicates its superiority over lower extremity muscles in diagnosing sarcopenia in male older Chinese adults. A recent study conducted among female rheumatoid arthritis patients in Japan reported an AUC of 0.809 for using biceps brachii CSA to predict sarcopenia [25], which was consistent with our finding. But it did not provide cut‐off values for low muscle mass. Another study found a strong correlation between biceps brachii CSA and lean mass measured by BIA, but also lacked specific cut‐off values [26]. For ultrasonography assessments of sarcopenia, lower extremity muscles are more frequently measured than upper extremity muscles, with the quadriceps being more commonly studied than the tibialis anterior [11]. Currently, there are no established cut‐off points for sarcopenia by comparing the diagnostic performance of upper limb versus lower limb muscles, with analyses differentiated by sex [11, 13]. Our study partially fills the gap and provides the optimal cut‐off values for ultrasound assessing low muscle mass in older Chinese adults.

Our study showed that low muscle mass as defined by biceps brachii CSA or tibialis anterior MT was associated with poor physical performance (as evaluated by SPPB, ADL and frailty), with the observed effect size being higher than that obtained through DXA or BIA. Additionally, inter‐rater reliability was also analysed. Biceps brachii CSA showed excellent inter‐rater reliability, surpassing other assessed parameters, and tibialis anterior MT showed good inter‐rater reliability. These findings provided solid evidence for the superior consistency and accuracy in these ultrasound measurements and the clinical application value in sarcopenia diagnosis.

Our observations substantiated the utility of MT and CSA for evaluation of muscle mass. Muscle thickness, muscle cross‐sectional area, fascicle length, echo‐intensity and pennation angle are commonly assessed parameters in the evaluation of muscle characteristics [8, 12]. The process of measuring pennation angle and fascicle length demands more complex methodologies compared with MT and CSA [27, 28, 29]. Our study exclusively concentrated on MT and CSA, given their apparent simplicity and efficiency as measurement tools [30]. These parameters offer a straightforward approach to quantifying muscle mass, which is particularly advantageous in clinical settings. Additionally, it is worth noting that shear‐wave elastography [31] and AI‐assisted muscle ultrasound [32], as relatively new techniques that assess muscle mass, were needed for future research to investigate the applicative potential in sarcopenia diagnosis.

Our study has several strengths. Firstly, a large sample size was specifically recruited from the seniors, with data analysed separately by sex. This accounted for sex differences and enhanced result reliability. Secondly, the optimal muscle sites and cut‐off values were determined from among seven candidate sites and 18 measurements. Thirdly, the inter‐rater reliability was assessed for ultrasound measurements, confirming the repeatability of the ultrasound examination process. Lastly, logistical regression verified the associations between muscle mass, defined by ultrasound thresholds, and adverse outcomes including SPPB, ADL and frailty, further indicating the utility of ultrasound diagnosis for muscle mass.

There were limitations to acknowledge. First, the sample population was primarily recruited from Zhejiang Province; this geographical restriction may limit the generalizability of the findings to broader populations, and future studies will be conducted to validate the applicability of these results in other provinces and ethnic or demographic groups. Second, being a cross‐sectional study, it lacked prospective results that could provide insights into how changes in muscle parameters correlate with physical outcomes over time. Future studies are needed to follow up on these changes to better understand the progression of ultrasound‐defined muscle mass and their impact on physical function. Third, the current study focused on inter‐rater reliability and did not assess intra‐rater reliability. While inter‐rater reliability provides valuable insights into the procedure's consistency across operators, future work incorporating intra‐rater reliability would strengthen the evidence for the temporal stability and reproducibility of our results. Fourth, assessments in hospitalized participants were conducted over two consecutive days. While minor day‐to‐day variations in hydration or physiological status could theoretically occur, such fluctuations are expected to have minimal impact on muscle measurements, particularly given the short interval and the stability of muscle parameters over brief periods. Therefore, these variations are unlikely to have significantly affected the main findings.

## 5 Conclusion

Our study provides robust supporting evidence for the use of ultrasound in diagnosing sarcopenia and establishes cut‐off values for older Chinese adults. Specifically, we demonstrated that the biceps brachii CSA in males and tibialis anterior MT in females is superior to other ultrasound parameters for predicting low muscle mass in this population. Biceps brachii CSA < 7.1 cm^2^ in males and tibialis anterior MT < 2.3 cm in females were identified as effective indicators for diagnosing low muscle mass. The diagnostic performances were comparable with BIA‐assessed ASMI when DXA‐defined ASMI is the standard. Low muscle mass defined by ultrasound was significantly associated with poor physical functions (physical performance, activities of daily life and frailty status), with the effect sizes higher than DXA or BIA‐defined low muscle mass. Additionally, the good inter‐rater reliability of these two parameters enhances their clinical applicability. In summary, our findings suggest that ultrasound is a reliable and practical tool for assessing low muscle mass in older adults, offering valuable reference values for the diagnosis of sarcopenia in clinical practice.

## Funding

The study was supported by the Key Research and Development Program of Zhejiang Province of China (2022C03161) and National Natural Science Foundation of China (82271588).

## Ethics Statement

The authors of this manuscript certify that they comply with the ethical guidelines for authorship and publishing in the *Journal of Cachexia, Sarcopenia and Muscle*. As reported in the ‘Methods’ section, this study has been approved by the ethics committee, being performed in accordance with the ethical standards laid down in the 1964 Declaration of Helsinki and its later amendments. All the participants gave their informed consent prior to their inclusion in the study.

## Conflicts of Interest

The authors declare no conflicts of interest.

## Supporting information


**Table S1:** Anatomical landmarks and ultrasound measurements for each muscle.
**Table S2:** The percentiles of ultrasound parameters for DXA‐defined low muscle mass. The diagnosis of low muscle mass was based on the revised criteria proposed by the Asian Working Group for Sarcopenia. According to dual‐energy X‐ray absorptiometry (DXA) measurements, low muscle mass was defined as an appendicular skeletal muscle mass index of less than 7.0 kg/m2 in men and less than 5.4 kg/m2 in women.
**Table S3:** The AUC of ultrasound parameters adjusted by height, height squared, weight and BMI for DXA‐defined low muscle mass.
**Table S4:** The mean standard error and minimal difference of measurements for reliability assessment.
**Figure S1:** Diagram of the ultrasound scanning procedure for different muscles. Ultrasound examinations were conducted when participants were positioned lying on the bed with their limbs fully relaxed and extended. Note: This section of the pictures is supplementary collection and not sourced from the participants.
**Figure S2:** The ultrasonic imagines for muscles. CSA: muscle cross‐sectional area, F: femur, FT: fat thickness, H: humerus, MT: muscle thickness, T: tibia.
**Figure S3:** Correlations between ultrasound measurements and age, BMI, DXA‐assessed ASMI, BIA‐assessed ASMI, grip strength and gait speed. Spearman test was performed to examine the bivariate correlation. CSA: cross‐sectional area, FT: fat thickness, MT: muscle thickness. *p < 0.05, **p < 0.01.
