# Correction to ‘Determination of Ultrasound Reference Values for Diagnosing Low Muscle Mass in Older Chinese Adults’

**DOI:** 10.1002/jcsm.70224

**Published:** 2026-02-19

S. Li , K. Xu , A. Wang , et al., “Determination of Ultrasound Reference Values for Diagnosing Low Muscle Mass in Older Chinese Adults,” Journal of Cachexia, Sarcopenia and Muscle 16, no. 6 (2025): e70155, 10.1002/jcsm.70155.41362106 PMC12686591

In the ‘Author List’ section, the fourth author's name was incorrectly listed as ‘Chenfan Qin’. This should have read: ‘Chengfan Qin’ and has now been corrected in the article.

We apologize for this error.

