# Supplementary material for: Determination of Ultrasound Reference Values for Diagnosing Low Muscle Mass in Older Chinese Adults
Source: J Cachexia Sarcopenia Muscle. 2025 Dec 8;16(6):e70155. doi: 10.1002/jcsm.70155 (PMC12686591; doi:10.1002/jcsm.70155)
Supplement: Supplementary file 1 — Table S1: Anatomical landmarks and ultrasound measurements for each muscle. Table S2: The percentiles of ultrasound parameters for DXA‐defined low muscle mass. The diagnosis of low muscle mass was based on the revised criteria proposed by the Asian Working Group for Sarcopenia. According to dual‐energy X‐ray absorptiometry (DXA) measurements, low muscle mass was defined as an appendicular skeletal muscle mass index of less than 7.0 kg/m2 in men and less than 5.4 kg/m2 in women. Table S3: The AUC of ultrasound parameters adjusted by height, height squared, weight and BMI for DXA‐defined low muscle mass. Table S4: The mean standard error and minimal difference of measurements for reliability assessment. Figure S1: Diagram of the ultrasound scanning procedure for different muscles. Ultrasound examinations were conducted when participants were positioned lying on the bed with their limbs fully relaxed and extended. Note: This section of the pictures is supplementary collection and not sourced from the participants. Figure S2: The ultrasonic imagines for muscles. CSA: muscle cross‐sectional area, F: femur, FT: fat thickness, H: humerus, MT: muscle thickness, T: tibia. Figure S3: Correlations between ultrasound measurements and age, BMI, DXA‐assessed ASMI, BIA‐assessed ASMI, grip strength and gait speed. Spearman test was performed to examine the bivariate correlation. CSA: cross‐sectional area, FT: fat thickness, MT: muscle thickness. *p < 0.05, **p < 0.01. [file JCSM-16-e70155-s001.doc]

**Supplementary material**

Supplementary Table 1. Anatomical landmarks and ultrasound measurements for each muscle.

|  | **Parameters** | **Proximal landmark** | **Distal landmark** | **Exact point** | **Remark** |
| --- | --- | --- | --- | --- | --- |
| Biceps brachii | Fat thickness | acromion | antecubital crease | The two-thirds | the dominant arm, at 45 degrees Angle to the body |
|  | Muscle thickness |  |  |  |  |
|  | Muscle cross sectional area |  |  |  |  |
|  | Pressed- muscle thickness |  |  |  |  |
| Rectus abdominis | Fat thickness | about 2-5 cm at the right of the umbilicus | **/** | The thickest point of muscle | at the end of inspiratory |
|  | Muscle thickness |  |  |  |  |
|  | Pressed- muscle thickness |  |  |  |  |
| Rectus femoris | Fat thickness | the anterior superior spine | the superior border of the patella | the midpoint | / |
|  | Muscle thickness |  |  |  |  |
|  | Muscle cross sectional area |  |  |  |  |
| Vastus intermedius | Muscle thickness | the anterior superior spine | the superior border of the patella | the midpoint | **/** |
| Quadriceps femoris | Pressed- muscle thickness | the anterior superior spine | the superior border of the patella | the midpoint | **/** |
| Vastus lateralis | Fat thickness | the anterior superior spine | the superior border of the patella | the two-thirds | **/** |
|  | Muscle thickness |  |  |  |  |
| Vastus medialis | Fat thickness | about 3 - 4 cm above the patella | **/** | The thickest point of muscle | **/** |
|  | Muscle thickness |  |  |  |  |
| Tibialis anterior | Fat thickness | the inferior border of the patella | the lateral malleolus | the upper one-quarter |  |
|  | Muscle thickness |  |  |  |  |

All images were collected at the transverse section, except for the vastus medialis at the coronal plane.

The fat thickness, muscle thickness and muscle cross sectional area were measured with the probe contacting skin without squeezing the muscle and subcutaneous fat. While the pressed- muscle thickness were measured with the probe pressed down on the body.

MT was the distance between deep and superficial aponeurosis.

CSA was the circumference of the muscle by manually drawing with a cursor, the mean value of two attempts was recorded.

**Supplementary Table 2. The percentiles of ultrasound parameters for DXA-defined low muscle mass.**

| **Ultrasound parameters** | **Normal muscle mass** | | | | |  | **Low muscle mass** | | | | |
| --- | --- | --- | --- | --- | --- | --- | --- | --- | --- | --- | --- |
| **Men** | **5th** | **25th** | **50th** | **75th** | **95th** |  | **5th** | **25th** | **50th** | **75th** | **95th** |
| Biceps brachii FT, cm | 0.06 | 0.17 | 0.25 | 0.34 | 0.51 |  | 0.05 | 0.13 | 0.21 | 0.32 | 0.48 |
| Biceps brachii MT, cm | 1.85 | 2.10 | 2.27 | 2.42 | 2.70 |  | 1.43 | 1.77 | 1.95 | 2.13 | 2.40 |
| Pressed-biceps brachii MT, cm | 1.39 | 1.76 | 1.98 | 2.27 | 2.63 |  | 1.04 | 1.41 | 1.65 | 1.91 | 2.19 |
| Biceps brachii CSA, cm^2^ | 5.85 | 7.20 | 7.97 | 8.98 | 10.62 |  | 4.00 | 5.38 | 6.21 | 7.23 | 8.43 |
| Rectus abdominis FT, cm | 0.62 | 1.43 | 1.79 | 2.27 | 3.10 |  | 0.47 | 1.02 | 1.45 | 1.95 | 2.74 |
| Rectus abdominis MT, cm | 0.64 | 0.81 | 0.93 | 1.06 | 1.25 |  | 0.54 | 0.68 | 0.82 | 0.92 | 1.09 |
| Pressed-rectus abdominis MT, cm | 0.50 | 0.65 | 0.74 | 0.84 | 1.08 |  | 0.43 | 0.54 | 0.63 | 0.72 | 0.85 |
| Rectus femoris FT, cm | 0.18 | 0.38 | 0.56 | 0.73 | 1.08 |  | 0.17 | 0.33 | 0.48 | 0.66 | 0.97 |
| Rectus femoris MT, cm | 1.25 | 1.49 | 1.67 | 1.81 | 2.04 |  | 0.88 | 1.18 | 1.40 | 1.60 | 1.87 |
| Vastus intermedius MT, cm | 0.86 | 1.20 | 1.44 | 1.70 | 2.11 |  | 0.60 | 0.85 | 1.01 | 1.34 | 1.69 |
| Quadriceps femoris MT, cm | 0.99 | 1.32 | 1.57 | 1.86 | 2.46 |  | 0.70 | 0.97 | 1.19 | 1.42 | 1.83 |
| Rectus femoris CSA, cm^2^ | 3.92 | 4.70 | 5.39 | 6.05 | 7.28 |  | 2.72 | 3.64 | 4.25 | 5.03 | 6.28 |
| Vastus lateralis FT, cm | 0.05 | 0.12 | 0.20 | 0.32 | 0.47 |  | 0.04 | 0.10 | 0.18 | 0.28 | 0.54 |
| Vastus lateralis MT, cm | 1.18 | 1.52 | 1.74 | 2.00 | 2.31 |  | 0.82 | 1.17 | 1.38 | 1.62 | 2.07 |
| Vastus medialis FT, cm | 0.10 | 0.25 | 0.39 | 0.50 | 0.75 |  | 0.08 | 0.24 | 0.37 | 0.51 | 0.72 |
| Vastus medialis MT, cm | 2.51 | 2.89 | 3.24 | 3.48 | 3.83 |  | 2.11 | 2.56 | 2.84 | 3.19 | 3.53 |
| Tibialis anterior FT, cm | 0.04 | 0.05 | 0.07 | 0.11 | 0.18 |  | 0.03 | 0.05 | 0.07 | 0.12 | 0.22 |
| Tibialis anterior MT, cm | 2.40 | 2.66 | 2.86 | 3.06 | 3.40 |  | 1.91 | 2.24 | 2.49 | 2.69 | 3.01 |
| **Women** |  |  |  |  |  |  |  |  |  |  |  |
| Biceps brachii FT, cm | 0.17 | 0.37 | 0.49 | 0.64 | 0.87 |  | 0.12 | 0.27 | 0.37 | 0.50 | 0.69 |
| Biceps brachii MT, cm | 1.31 | 1.56 | 1.70 | 1.85 | 2.05 |  | 1.10 | 1.39 | 1.52 | 1.64 | 1.85 |
| Pressed-biceps brachii MT, cm | 0.99 | 1.24 | 1.48 | 1.65 | 1.93 |  | 0.83 | 0.98 | 1.20 | 1.41 | 1.70 |
| Biceps brachii CSA, cm^2^ | 3.39 | 4.33 | 4.90 | 5.66 | 6.57 |  | 2.69 | 3.57 | 3.97 | 4.49 | 5.35 |
| Rectus abdominis FT, cm | 0.85 | 1.98 | 2.51 | 3.11 | 4.08 |  | 0.80 | 1.40 | 2.04 | 2.61 | 3.60 |
| Rectus abdominis MT, cm | 0.44 | 0.56 | 0.67 | 0.75 | 0.95 |  | 0.35 | 0.48 | 0.60 | 0.69 | 0.96 |
| Pressed-rectus abdominis MT, cm | 0.35 | 0.47 | 0.54 | 0.61 | 0.78 |  | 0.31 | 0.41 | 0.49 | 0.58 | 0.74 |
| Rectus femoris FT, cm | 0.38 | 0.72 | 0.94 | 1.20 | 1.65 |  | 0.33 | 0.54 | 0.80 | 1.06 | 1.56 |
| Rectus femoris MT, cm | 1.00 | 1.25 | 1.41 | 1.57 | 1.82 |  | 0.75 | 1.02 | 1.18 | 1.36 | 1.62 |
| Vastus intermedius MT, cm | 0.62 | 0.95 | 1.19 | 1.42 | 1.80 |  | 0.38 | 0.69 | 0.87 | 1.10 | 1.41 |
| Quadriceps femoris MT, cm | 0.72 | 1.01 | 1.22 | 1.44 | 1.79 |  | 0.45 | 0.68 | 0.89 | 1.10 | 1.46 |
| Rectus femoris CSA, cm^2^ | 2.79 | 3.42 | 3.93 | 4.65 | 6.03 |  | 1.99 | 2.86 | 3.16 | 3.86 | 4.57 |
| Vastus lateralis FT, cm | 0.14 | 0.34 | 0.56 | 0.78 | 1.15 |  | 0.08 | 0.29 | 0.53 | 0.74 | 0.96 |
| Vastus lateralis MT, cm | 0.89 | 1.26 | 1.51 | 1.72 | 2.06 |  | 0.60 | 0.92 | 1.16 | 1.46 | 1.79 |
| Vastus medialis FT, cm | 0.37 | 0.55 | 0.74 | 0.94 | 1.31 |  | 0.31 | 0.48 | 0.65 | 0.78 | 1.09 |
| Vastus medialis MT, cm | 2.26 | 2.57 | 2.81 | 3.04 | 3.47 |  | 1.94 | 2.31 | 2.54 | 2.74 | 3.08 |
| Tibialis anterior FT, cm | 0.05 | 0.10 | 0.16 | 0.25 | 0.45 |  | 0.04 | 0.07 | 0.16 | 0.24 | 0.37 |
| Tibialis anterior MT, cm | 1.98 | 2.30 | 2.46 | 2.63 | 2.91 |  | 1.62 | 1.90 | 2.11 | 2.30 | 2.56 |

DXA: Dual-energy X-ray absorptiometry, FT: fat thickness, MT: muscle thickness, CSA: muscle cross sectional area.

The diagnosis of low muscle mass was based on the revised criteria proposed by the Asian Working Group for Sarcopenia. According to dual-energy X-ray absorptiometry (DXA) measurements, low muscle mass was defined as an appendicular skeletal muscle mass index of less than 7.0 kg/m² in men and less than 5.4 kg/m² in women.

**Supplementary Table 3. The AUC of ultrasound parameters adjusted by height, height squared, weight and BMI for DXA-defined low muscle mass.**

|  | **Height-adjusted** | | |  | **Height squared-adjusted** | | |  | **Weight-adjusted** | | |  | **BMI-adjusted** | | |
| --- | --- | --- | --- | --- | --- | --- | --- | --- | --- | --- | --- | --- | --- | --- | --- |
|  | **AUC** | **95%CI** | |  | **AUC** | **95%CI** | |  | **AUC** | **95%CI** | |  | **AUC** | **95%CI** | |
| **Men** |  | **Lower** | **Upper** |  |  | **Lower** | **Upper** |  |  | **Lower** | **Upper** |  |  | **Lower** | **Upper** |
| Biceps brachii MT, cm | 0.777 | 0.731 | 0.822 |  | 0.738 | 0.689 | 0.786 |  | 0.459 | 0.403 | 0.514 |  | 0.502 | 0.447 | 0.558 |
| Pressed-biceps brachii MT, cm | 0.745 | 0.697 | 0.794 |  | 0.724 | 0.674 | 0.774 |  | 0.527 | 0.471 | 0.583 |  | 0.556 | 0.500 | 0.611 |
| Biceps brachii CSA, cm^2^ | **0.822** | 0.781 | 0.864 |  | **0.801** | 0.758 | 0.844 |  | 0.626 | 0.572 | 0.681 |  | **0.657** | 0.604 | 0.710 |
| Rectus abdominis MT, cm | 0.695 | 0.643 | 0.747 |  | 0.672 | 0.619 | 0.725 |  | 0.477 | 0.420 | 0.534 |  | 0.505 | 0.448 | 0.562 |
| Pressed-rectus abdominis MT, cm | 0.710 | 0.659 | 0.761 |  | 0.687 | 0.635 | 0.739 |  | 0.488 | 0.431 | 0.545 |  | 0.519 | 0.462 | 0.577 |
| Rectus femoris MT, cm | 0.742 | 0.694 | 0.790 |  | 0.717 | 0.668 | 0.767 |  | 0.493 | 0.438 | 0.549 |  | 0.531 | 0.476 | 0.586 |
| Vastus intermedius MT, cm | 0.762 | 0.715 | 0.808 |  | 0.751 | 0.703 | 0.799 |  | 0.628 | 0.574 | 0.683 |  | 0.650 | 0.597 | 0.703 |
| Quadriceps femoris MT, cm | 0.777 | 0.732 | 0.822 |  | 0.765 | 0.719 | 0.812 |  | **0.631** | 0.577 | 0.685 |  | 0.653 | 0.600 | 0.706 |
| Rectus femoris CSA, cm^2^ | 0.763 | 0.717 | 0.809 |  | 0.746 | 0.699 | 0.794 |  | 0.569 | 0.513 | 0.624 |  | 0.597 | 0.542 | 0.652 |
| Vastus lateralis MT, cm | 0.750 | 0.703 | 0.798 |  | 0.731 | 0.683 | 0.780 |  | 0.557 | 0.501 | 0.612 |  | 0.592 | 0.537 | 0.647 |
| Vastus medialis MT, cm | 0.690 | 0.637 | 0.742 |  | 0.659 | 0.605 | 0.713 |  | 0.379 | 0.324 | 0.435 |  | 0.421 | 0.365 | 0.477 |
| Tibialis anterior MT, cm | 0.787 | 0.742 | 0.832 |  | 0.746 | 0.697 | 0.795 |  | 0.423 | 0.368 | 0.478 |  | 0.464 | 0.408 | 0.519 |
| **Women** |  |  |  |  |  |  |  |  |  |  |  |  |  |  |  |
| Biceps brachii MT, cm | 0.698 | 0.638 | 0.758 |  | 0.656 | 0.592 | 0.719 |  | 0.340 | 0.274 | 0.406 |  | 0.376 | 0.309 | 0.444 |
| Pressed-biceps brachii MT, cm | 0.711 | 0.651 | 0.772 |  | 0.692 | 0.631 | 0.753 |  | 0.465 | 0.393 | 0.536 |  | 0.507 | 0.434 | 0.580 |
| Biceps brachii CSA, cm^2^ | 0.776 | 0.724 | 0.827 |  | 0.757 | 0.703 | 0.811 |  | 0.512 | 0.441 | 0.582 |  | 0.549 | 0.479 | 0.619 |
| Rectus abdominis MT, cm | 0.601 | 0.532 | 0.670 |  | 0.581 | 0.511 | 0.650 |  | 0.391 | 0.323 | 0.460 |  | 0.412 | 0.344 | 0.481 |
| Pressed-rectus abdominis MT, cm | 0.582 | 0.512 | 0.651 |  | 0.562 | 0.492 | 0.631 |  | 0.359 | 0.292 | 0.426 |  | 0.388 | 0.321 | 0.456 |
| Rectus femoris MT, cm | 0.728 | 0.668 | 0.788 |  | 0.707 | 0.646 | 0.768 |  | 0.446 | 0.377 | 0.515 |  | 0.485 | 0.415 | 0.556 |
| Vastus intermedius MT, cm | 0.737 | 0.680 | 0.795 |  | 0.726 | 0.667 | 0.785 |  | **0.591** | 0.520 | 0.662 |  | **0.612** | 0.543 | 0.682 |
| Quadriceps femoris MT, cm | 0.748 | 0.690 | 0.807 |  | 0.736 | 0.676 | 0.797 |  | 0.586 | 0.513 | 0.658 |  | **0.612** | 0.542 | 0.683 |
| Rectus femoris CSA, cm^2^ | 0.732 | 0.672 | 0.791 |  | 0.712 | 0.651 | 0.773 |  | 0.496 | 0.430 | 0.562 |  | 0.524 | 0.458 | 0.590 |
| Vastus lateralis MT, cm | 0.713 | 0.653 | 0.774 |  | 0.702 | 0.640 | 0.764 |  | 0.526 | 0.455 | 0.596 |  | 0.562 | 0.491 | 0.632 |
| Vastus medialis MT, cm | 0.696 | 0.635 | 0.758 |  | 0.658 | 0.595 | 0.722 |  | 0.318 | 0.258 | 0.379 |  | 0.348 | 0.283 | 0.412 |
| Tibialis anterior MT, cm | **0.815** | 0.766 | 0.864 |  | **0.771** | 0.717 | 0.826 |  | 0.394 | 0.325 | 0.463 |  | 0.430 | 0.360 | 0.501 |

The AUC values for ultrasound parameters adjusted for height, height squared, weight, and BMI, which were calculated by dividing the ultrasound parameters by these variables, respectively. The Bold indicates the maximum AUC values for different sex. FT: fat thickness, MT: muscle thickness, CSA: muscle cross sectional area, AUC: Area under the Curve.

**Supplementary Table 4. The mean standard error and minimal difference of measurements for reliability assessment.**

| **Measurements** | **Rater 1** | | **Rater 2** | | **MSE within raters** | | **Between raters** | |
| --- | --- | --- | --- | --- | --- | --- | --- | --- |
|  | **Mean** | **SD** | **Mean** | **SD** | **Rater 1** | **Rater 2** | **MSE** | **MD** |
| Biceps brachii MT, cm | 1.794 | 0.420 | 1.802 | 0.350 | 0.043 | 0.036 | 0.026 | 0.071 |
| Pressed-biceps brachii MT, cm | 1.310 | 0.397 | 1.484 | 0.353 | 0.040 | 0.036 | 0.029 | 0.080 |
| Biceps brachii CSA, cm^2^ | 6.084 | 2.048 | 5.511 | 1.708 | 0.210 | 0.175 | 0.084 | 0.232 |
| Rectus abdominis MT, cm | 0.783 | 0.204 | 0.735 | 0.198 | 0.021 | 0.020 | 0.013 | 0.035 |
| Pressed-rectus abdominis MT, cm | 0.615 | 0.162 | 0.584 | 0.154 | 0.016 | 0.016 | 0.009 | 0.025 |
| Rectus femoris MT, cm | 1.132 | 0.287 | 1.312 | 0.270 | 0.029 | 0.028 | 0.028 | 0.078 |
| Vastus intermedius MT, cm | 1.014 | 0.299 | 1.025 | 0.335 | 0.031 | 0.034 | 0.024 | 0.066 |
| Quadriceps femoris MT, cm | 1.079 | 0.354 | 1.076 | 0.334 | 0.036 | 0.034 | 0.021 | 0.057 |
| Rectus femoris CSA, cm^2^ | 3.850 | 1.223 | 3.994 | 1.049 | 0.123 | 0.106 | 0.100 | 0.276 |
| Vastus lateralis MT, cm | 1.636 | 0.363 | 1.426 | 0.382 | 0.037 | 0.039 | 0.037 | 0.103 |
| Vastus medialis MT, cm | 3.274 | 0.602 | 2.846 | 0.403 | 0.061 | 0.041 | 0.051 | 0.142 |
| Tibialis anterior MT, cm | 2.346 | 0.368 | 2.405 | 0.390 | 0.037 | 0.039 | 0.028 | 0.078 |

The mean standard errors were analyzed using paired sample t test. MD = SEM*1.96*√2. FT: fat thickness, MT: muscle thickness, CSA: muscle cross sectional area, SD: standard deviation, MSE: mean standard error, MD: minimal difference.


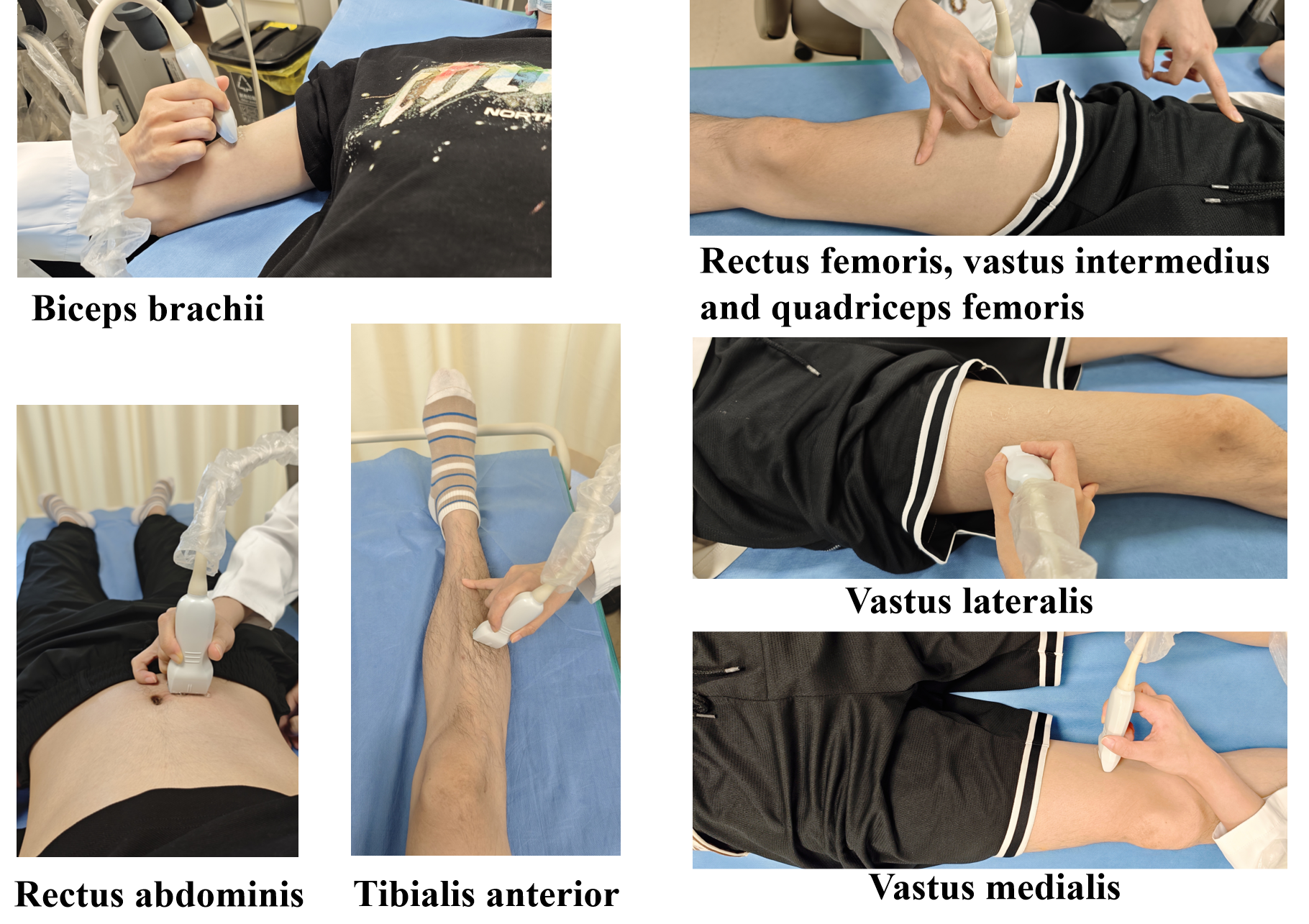


**Supplementary Figure 1. Diagram of the ultrasound scanning procedure** **for different muscles.**

Ultrasound examinations were conducted when participants were positioned lying on the bed with their limbs fully relaxed and extended.

Note: This section of the pictures is supplementary collection and not sourced from the participants.


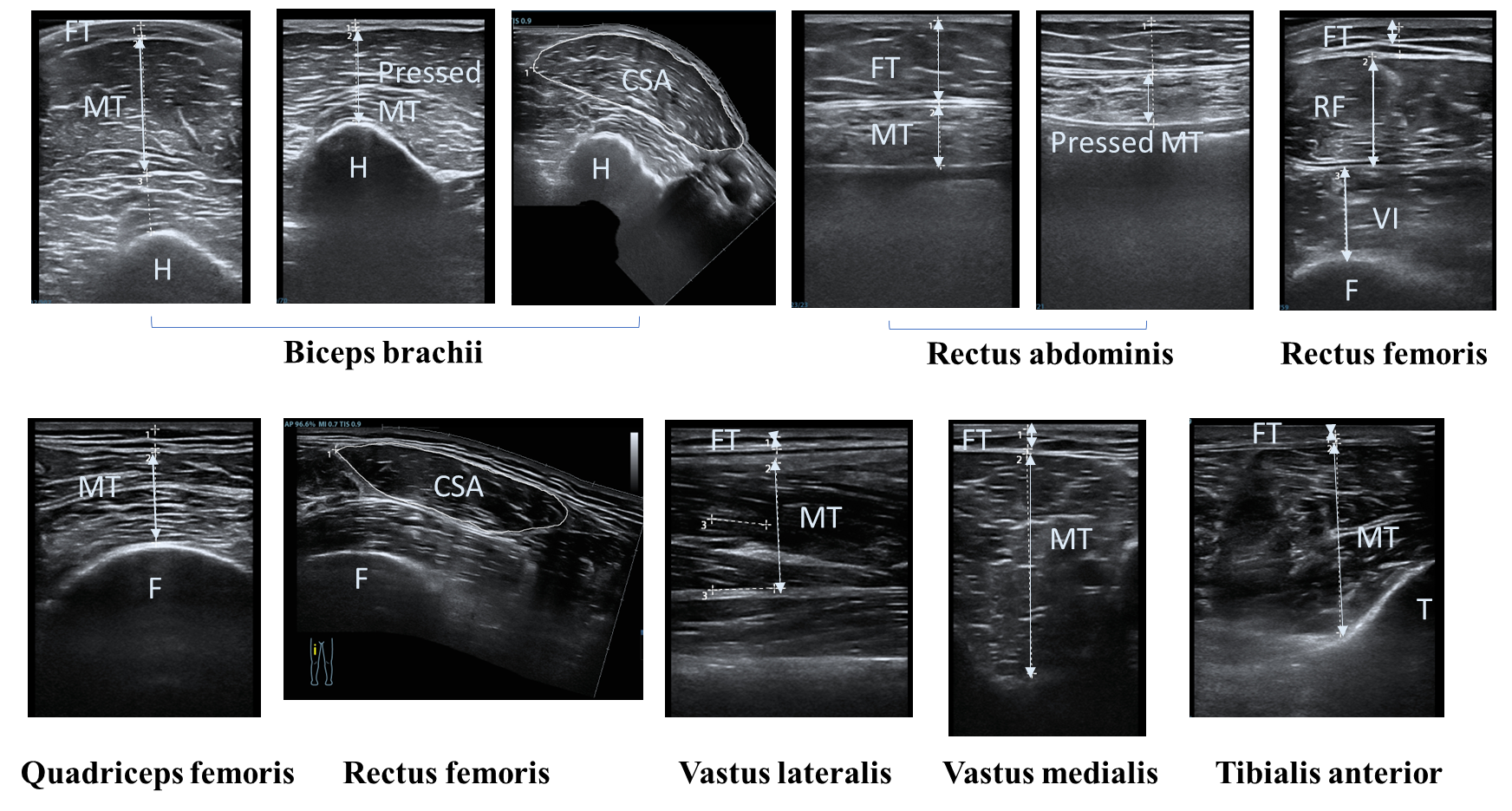


**Supplementary Figure 2. The ultrasonic imagines for muscles.**

FT: fat thickness, MT: muscle thickness, CSA: muscle cross sectional area. H: humerus, F: femur, T: tibia.


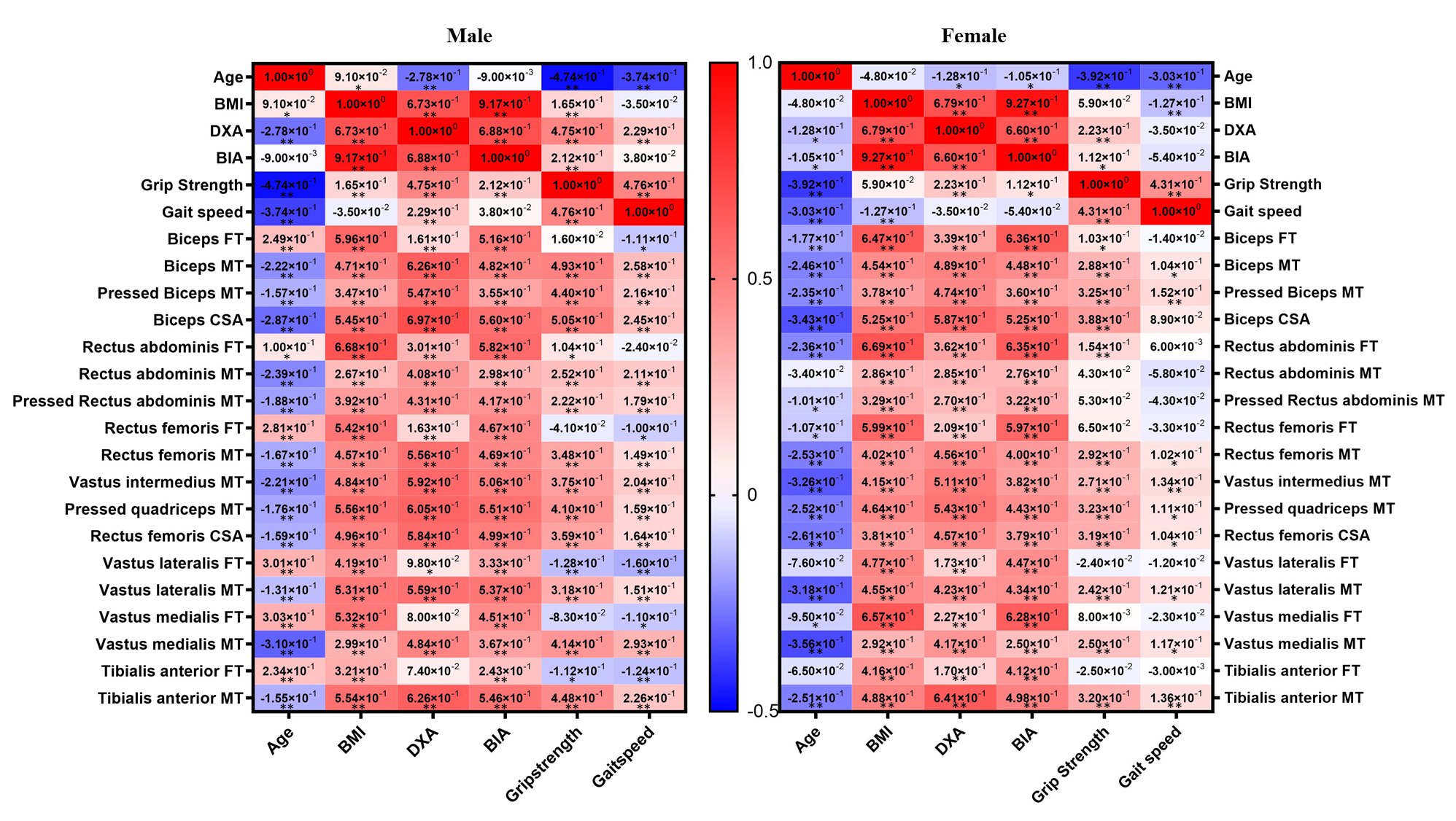


**Supplementary Figure 3. Correlations between ultrasound measurements and age, BMI, DXA-assessed ASMI, BIA-assessed ASMI, grip strength and gait speed.**

Spearman test was performed to examine the bivariate correlation. FT: fat thickness, MT: muscle thickness, CSA: cross sectional area. * p<0.05, ** p<0.01.
